# What Story Does Geographic Separation of Insular Bats Tell? A Case Study on Sardinian Rhinolophids

**DOI:** 10.1371/journal.pone.0110894

**Published:** 2014-10-23

**Authors:** Danilo Russo, Mirko Di Febbraro, Hugo Rebelo, Mauro Mucedda, Luca Cistrone, Paolo Agnelli, Pier Paolo De Pasquale, Adriano Martinoli, Dino Scaravelli, Cristiano Spilinga, Luciano Bosso

**Affiliations:** 1 Wildlife Research Unit, Dipartimento di Agraria, Università degli Studi di Napoli Federico II, Portici, Napoli, Italy; 2 School of Biological Sciences, University of Bristol, Bristol, United Kingdom; 3 EnvixLab, Dipartimento Bioscienze e Territorio, Università del Molise, Pesche, Italy; 4 CIBIO, Centro de Investigação em Biodiversidade e Recursos Genéticos da Universidade do Porto, University of Porto, Vairão, Portuga; 5 Centro per lo studio e la protezione dei pipistrelli in Sardegna, Sassari, Italy; 6 Forestry and Conservation, Cassino, Frosinone, Italy; 7 Museo di Storia Naturale dell’Università di Firenze, Sezione di Zoologia ‘La Specola’, Firenze, Italy; 8 Wildlife Consulting, Palo del Colle, Bari, Italy; 9 Unità di Analisi e Gestione delle Risorse Ambientali, Guido Tosi Research Group, Dipartimento di Scienze Teoriche e Applicate, Università degli Studi dell’Insubria, Varese, Italy; 10 Dipartimento di Scienze Mediche Veterinarie, Università degli Studi di Bologna, Ozzano dell’Emilia, Bologna, Italy; 11 Studio Naturalistico Hyla snc, Tuoro sul Trasimeno, Perugia, Italy; University of Regina, Canada

## Abstract

Competition may lead to changes in a species’ environmental niche in areas of sympatry and shifts in the niche of weaker competitors to occupy areas where stronger ones are rarer. Although mainland Mediterranean (*Rhinolophus euryale*) and Mehely’s (*R. mehelyi*) horseshoe bats mitigate competition by habitat partitioning, this may not be true on resource-limited systems such as islands. We hypothesize that Sardinian *R. euryale* (SAR) have a distinct ecological niche suited to persist in the south of Sardinia where *R. mehelyi* is rarer. Assuming that SAR originated from other Italian populations (PES) – mostly allopatric with *R. mehelyi* – once on Sardinia the former may have undergone niche displacement driven by *R. mehelyi*. Alternatively, its niche could have been inherited from a Maghrebian source population. We: a) generated Maxent Species Distribution Models (SDM) for Sardinian populations; b) calibrated a model with PES occurrences and projected it to Sardinia to see whether PES niche would increase *R. euryale*’s sympatry with *R. mehelyi*; and c) tested for niche similarity between *R. mehelyi* and PES, PES and SAR, and *R. mehelyi* and SAR. Finally we predicted *R. euryale*’s range in Northern Africa both in the present and during the Last Glacial Maximum (LGM) by calibrating SDMs respectively with SAR and PES occurrences and projecting them to the Maghreb. *R. mehelyi* and PES showed niche similarity potentially leading to competition. According to PES’ niche, *R. euryale* would show a larger sympatry with *R. mehelyi* on Sardinia than according to SAR niche. Such niches have null similarity. The current and LGM Maghrebian ranges of *R. euryale* were predicted to be wide according to SAR’s niche, negligible according to PES’ niche. SAR’s niche allows *R. euryale* to persist where *R. mehelyi* is rarer and competition probably mild. Possible explanations may be competition-driven niche displacement or Maghrebian origin.

## Introduction

Species distribution patterns may potentially result from a range of causes, historical or current, involving abiotic factors as well as biotic interactions [1]. Identifying which factors determine species distribution among the several potential candidates may not be obvious.

A paradigm of ecology is that long-term coexistence is impossible for species sharing an identical ecological niche due to competitive and stochastic factors [2]–[4]. Opposite evolutionary pressures may act on sympatric species in the same guild. Ecomorphological convergence may take place as a result of selective pressures associated with optimal exploitation of the same resources; on the other hand, if such resources are limiting, interspecific competition may occur, leading to niche segregation.

Several types of such mechanisms have been described, including spatial or temporal niche separation [5]–[7] and resource partitioning by morphological divergence [8]–[10]. Interspecific competition may lead to ecological character displacement: differences in morphological and behavioural traits between species are greater where the latter occur in sympatry, smaller or absent in allopatric conditions [2], [11], [12]. Character displacement is often referred to morphological divergence, whose relationship with resource utilization may sometimes be questionable [13], [14]. However, other functionally important traits characterizing a species’ ecological niche may undergo displacement, with crucial consequences for geographical distribution. Yet, the spatial dimension of competition – i.e., the large-scale alteration of species distribution due to biotic interactions – is a poorly explored issue. Interspecific competition might involve changes in the environmental niche of a species where the latter is sympatric to competitors, a process hereafter termed as “niche displacement” [14]. Assessing niche displacement constitutes a key approach to a better understanding of factors influencing species’ geographical range and niche features, offering a major insight into present and future distributional dynamics [14]. Clearer patterns are expected where competition is especially harsh. This is the case with insular environments, where resources are often limiting [15]: thus, islands provide an ideal set to study these processes.

However, caution is needed when interpreting the current characteristics of the ecological niche: rather than resulting from forces acting in situ, they could have been shaped by historical processes occurred ex situ, i.e. in the population’s geographical source, and then retained by their descendents in the newly established population (such as in a process of island colonization from the mainland). Although the rapid change of ecological traits have attracted the attention of scientists for centuries, there is increasing evidence that the tendency for many ecological traits to be retained over time, called niche conservatism [16] is an important, general phenomenon with major evolutionary and ecological consequences – among which, the stability of species assemblages [17].

Bats represent interesting models to test the effect of interactions between species that share habitats and ecomorphological traits due to adaptive convergence, phylogenetic relatedness or crypticism [18]–[20]. Although among bats several examples of niche segregation due to divergence in morphology, sensory ecology, foraging strategies or habitat partitioning are known [21]–[24], no evidence of competition-driven geographical displacement is available.

In this study we focus on two rhinolophid bat species, the Mediterranean (*Rhinolophus euryale*) and Mehely’s (*Rhinolophus mehelyi*) horseshoe bats. These largely sympatric Mediterranean bats [25] may be regarded as sibling species as they derive from a close common ancestor and are morphologically very similar [26], [27]. They are thought to have diverged only 3 My ago [26] and only in 1901 were they recognized as separate species by the German zoologist Paul Matschie. *R. euryale* is widely distributed from sea level to ca. 1,000 m a.s.l. in the south of the continent as well as north-west Africa, and the Near East [28]. Classified globally as near threatened, *R. euryale* populations are declining in most of the geographical range [28]. *R. mehelyi* is confined to the Mediterranean where it shows a patchy occurrence from north Africa and southern Europe through Asia Minor, Anatolia, to Transcaucasia, Iran and Afghanistan [29]. The species is classified as vulnerable on a global scale, and is reported to be extinct in north-east Spain, Mallorca [30], Croatia and Israel [31] and close to extinction in France [32] and Romania [33].

These species have been regarded as potential competitors when foraging in sympatry for marked similarities in morphology, echolocation and habitat selection [34]–[36] yet, provided environmental conditions are sufficiently heterogeneous, they may mitigate competition by fine-scale habitat partitioning [35], [36].

The Italian distribution of these bats is puzzling. *R. mehelyi* is frequent and relatively abundant on Sardinia, whereas in the rest of Italy is almost absent – in fact on the brink of extinction, being restricted to two sites in Sicily where only small colonies occur, and one site on the mainland (Apulia, south-east Italy) where only in 2013 was a single individual observed after 40 years since the latest sighting [37]. *R. euryale* is widespread in most of the Italian peninsula and also occurs in Sicily. On Sardinia, although both species are present, *R. mehelyi* occurs in allopatry in most of the island while their sympatry is restricted to a small area. There, the two species show divergence in echolocation call frequency [38]. Specifically, Sardinian *R. euryale* shows lower frequencies than the peninsular conspecifics, a difference thought to represent an acoustic character displacement pattern driven by the dominant *R. mehelyi* probably to avoid interspecific frequency overlap and maintain separate communication frequency bandwidths [38].

Islands are ecological systems where spatial and trophic resources are often limited and may lead to increased competition [39], [40]. In this study we used distributional data, maximum entropy models (Maxent) and Niche Analysis to test the main hypothesis that in an insular, food-limited environment (Sardinia), *R. euryale* may have at least partly accomplished geographical separation from its sibling species thanks to a distinct ecological niche which has allowed it to settle in an area where *R. mehelyi* is rare and competition probably negligible.

This hypothesis generates two predictions:

- Significant overlap will occur between the environmental niches of Sardinian *R. mehelyi* and allopatric *R. euryale* populations from the mainland and Sicily (hereafter termed PES), setting the scene for interspecific competition;
- although conspecifics, the niche of Sardinian *R. euryale* (hereafter termed SAR) will diverge from that of PES. This divergence will allow SAR to mitigate interspecific competition with *R. mehelyi*.

Assuming that SAR has originated from PES, once bats colonized Sardinia the original ecological niche may have undergone a niche displacement process driven by *R. mehelyi’s* competition and generated the difference forecast by prediction b). However, the origin of *R. euryale’s* population on Sardinia is unknown. Along with Europe, northern Africa represents an important geographical source for Sardinian bats [41]–[43]. Under a niche conservatism assumption [16], any niche difference spotted in SAR relative to PES might rather represent a legacy of an extra-European source population which colonized Sardinia and founded SAR. It may be hypothesized that once bats colonized the island, they retained their ecological niche by stabilizing selection [44] because it performed well in the southern region of Sardinia where competition with *R. mehelyi* was limited.

Accordingly, to search for clues on SAR’s origin, we tested whether SAR’s niche would perform better than PES’ niche in the Maghrebian geographical set. This prediction would be consistent with a northern African origin of SAR. We tested this under different temporal scenarios: we trained distribution models with SAR and PES occurrences respectively and projected them to northern Africa in two snapshots – current time and Last Glacial Maximum (LGM, 21,000 years PB). We chose LGM because at that time geographical distances between islands and mainland were reduced by the emergence of land bridges favouring island colonization by bats, including that of Sardinia from northern Africa [42], [45].

## Materials and Methods

### Study area

For this study we considered the entire Italian territory comprised ca. between latitudes 45° N–36° N and longitudes 6°E–18°E (corresponding to ca. 301.000 km^2^, elevation range = 0–4810 m a.s.l.).

### Presence species data

**Figure 1:**
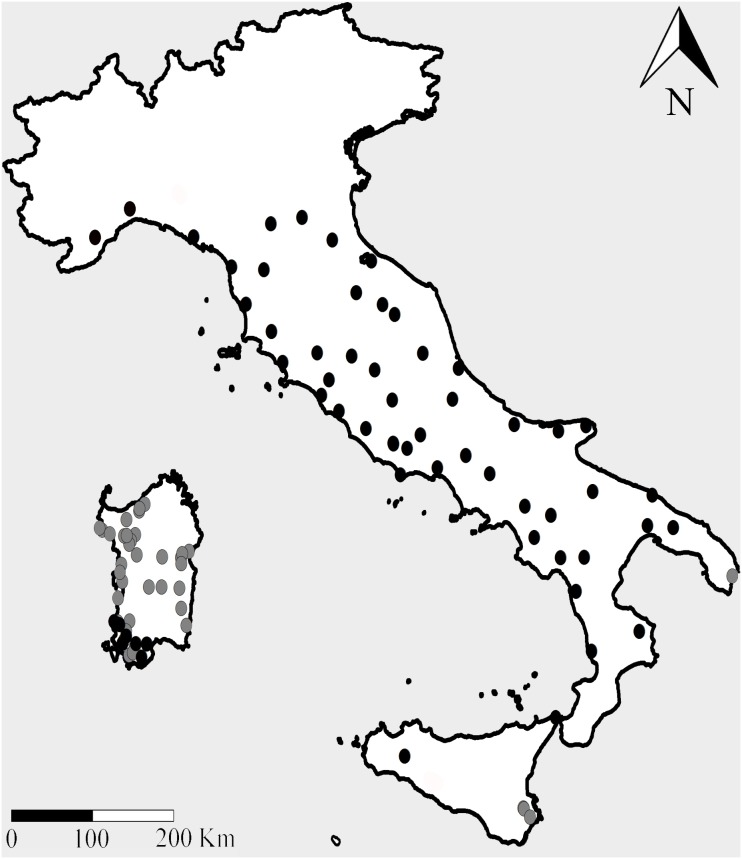
Presence records for *Rhinolophus euryale* (n = 65; black symbols) and *R. mehelyi* (n = 40; grey symbols) considered for this study. The publicly available map layer was obtained from www.fao.org/geonetwork/srv/en/main.home and the image prepared with the Quantum Gis 2.2.0 Valmiera open source software.

Presence records for *R. euryale* (n = 210) and *R. mehelyi* (n = 60) came from authors’ personal databases (Figure 1). Most faunal records were taken by either direct observation or acoustic surveys – activities requiring no specific permission according to Italian laws and regulations. On Sardinia, when roosts were surveyed for the first time the distinction between *R. euryale* and *R. mehelyi* was made by temporarily capturing bats under licence from the Italian Ministry of Environment (licence numbers: DPN/2D/2004/7489, DPN-2007-0003938, DPN-2010-0009609). Records were screened in ArcGis (version 9.2) for spatial autocorrelation using average nearest neighbour analyses and Moran’s I measure of spatial autocorrelation to remove spatially correlated data points and guarantee independence. After this selection, 65 and 40 presence data respectively for *R. euryale* and *R. mehelyi* were used to generate SDMs.

### Ecogeographical variables

To predict habitat suitability for the two species, we used a set of 21 Eco-Geographical Variables (EGVs). We included one topographical and 19 bioclimatic variables obtained from WorldClim database (www.worldclim.org/current) [46]. The latter variables are derived from the monthly temperature and rainfall values in order to generate more biologically meaningful factors [47]. Land cover was obtained from Global Land Cover 2000 (http://bioval.jrc.ec.europa.eu/products/glc2000/products.php). All variable formats were raster files (grid) with a resolution of 30 arc second (0.93×0.93 km = 0.86 km^2^ at the equator) and 1,307,195 grid cells. In order to remove the highly correlated variables for the final distribution models, we calculated a correlation matrix using Pearson’s technique and selected only the variables with r<0.5. We converted the eleven final EGVs used to model habitat suitability of both species in ASCII files.

### Maximum entropy approach

We used Maxent – maximum entropy modelling of species geographic distributions [48] – to develop a geographic distribution model for *R. euryale* and *R. mehelyi*. Maxent is a machine learning method developed to detect habitat suitability of each grid cell as function of the interaction between EGVs and occurrence data [48]. This approach does not require absence data to model, an especially important feature for nocturnal, elusive animals such as bats. To build the models, we used Maxent ver. 3.3.3 k (http://www.cs.princeton.edu/~schapire/maxent), the presence record for *R. euryale* and *R. mehelyi* selected as described above, and the following EGVs: Altitude, Land cover, Mean Diurnal Range, Isothermality, Temperature Seasonality, Temperature Annual Range, Mean Temperature of Wettest Quarter, Mean Temperature of Driest Quarter, Precipitation Seasonality, Precipitation of Wettest Quarter and Precipitation of Coldest Quarter. Further details on EGV are given in Table S1. In the setting panel, we selected the following options: random seed; remove duplicate presence records; write plot data; regularisation multiplier (fixed at 1); 10,000 maximum number of background points; 1000 maximum iterations; and, finally, 20 replicate effects with cross-validate replicated run type. For the latter procedure, 80% of records were randomly extracted for training and 20% for testing the model and the procedure was repeated 20 times. The average final map obtained had a logistic output format with suitability values from 0 (unsuitable habitat) to 1 (suitable habitat). The 10th percentile (the value above which the model classifies correctly 90% of the training locations) was selected as the threshold value for defining the species’ presence. This is a conservative value that is commonly used in species distribution modelling studies especially when considering datasets gathered over a long time by different observers and methods of collection. This threshold was used to reclassify our model into binary presence/absence maps [49].

We used Jacknife analysis to estimate the actual contribution that each variable provided to the geographic distribution models. During this process, Maxent generated three models: first, each EGV was excluded in turn and a model created with the remaining variables to check which of the latter was most informative. Second, a model was created using individually each EGV to detect which variable had the most information not featuring in the others. Third, a final model was generated based on all variables. Response curves derived from univariate models were plotted to know how each EGV influences the presence probability.

For *R. mehelyi* we generated a distribution model based on Sardinian occurrences only. For *R. euryale*, we generated two models: one calibrated with Sardinian occurrences only and projected to Sardinia (SAR), another based on occurrences from both the Italian peninsula and Sicily (PES) which was also projected to Sardinia. We also generated palaeo-distribution models based on bioclimatic variables only. These were trained with PES and SAR occurrences and projected to the Maghreb in the LGM (23,000–18,000 year BP). The two LGM models were based respectively on the Community Climate System Model, CCSM, and the Model for Interdisciplinary Research on Climate, MIROC [46], [50]. Projecting SDMs to regions other than those on which models were calibrated, or to past or future times is a widespread approach to make inferences such as forecasting the spreading of alien organisms, providing palaeo-reconstructions or predicting distributional patterns in future epochs [51]–[53]. In order to project to a new area models calibrated elsewhere, whether in the current epoch or in the LGM, variables in the projection area must meet a condition of environmental similarity to the environmental data used for training the model. Therefore, we preliminarily ascertained that this condition was verified for both current and past projections, which were thus legitimate, by inspecting Multivariate Environmental Similarity Surfaces [54] (data not shown in the results for brevity). All digital information had a resolution of 2.5 arc-minutes (4.6 km).

### Model validation

We evaluated model performance with different methods: the receiver operated characteristics (ROC), analyzing the area under curve (AUC) [55]; the true skill statistic (TSS) [56]; and the minimum difference between training and test AUC data (AUC_diff_) [57]. Such statistics were averaged across the 20 replicates run on the 80% (training) vs. 20% (testing) dataset split.

AUC established the discrimination ability of the models and may range from 0 (equalling random distribution) to 1 (perfect prediction). AUC values >0.75 correspond to high discrimination performances [58]. TSS compares the number of correct forecasts, minus those attributable to random guessing, to that of a hypothetical set of perfect forecasts. It considers both omission and commission errors, and success as a result of random guessing, and ranges from − 1 to +1, where +1 indicates perfect agreement and values of zero or less correspond to a performance no better than random [56]. By minimizing the difference between training and test AUC data, in fact we reduce the risk that models are over-parameterized in such a way as to be overly specific to the training data [57].

### Niche analysis

We performed niche overlap analyses using the analytical framework proposed by [59] and recently adopted in different studies [60], [61]. The procedure follows three steps: data pre-processing, calculation of the niche overlap measure and testing niche similarity. Further details are given in file S1.

To quantify niche overlap, we used the following ordination (for details see [59]) and SDMs methods: Principal Component Analysis calibrated with EGV values associated with the occurrences of the species (PCA-occ); Principal Component Analysis calibrated on the whole environmental space including the presence records where the species occur (PCA-env); Between-group and Within-group analyses (BETWEEN-occ and WITHIN-occ); Within-group calibrated on the whole environmental space (WITHIN-env); Linear Discriminant Analysis (LDA); Multidimensional scaling (MDS); and Maximum Entropy algorithm (MAXENT). For the application of the latter, two tests were carried out (named Maxent 1 and 2) corresponding to the analysis made using in turn one of the two Maxent outputs generated by either population (or species) as the comparison background against which the output of the remainder was contrasted.

## Results

### Niche differences between *R. mehelyi*, SAR and PES

Maxent models showed high levels of predictive performance as can be seen from AUC, TSS and AUC_diff_ values (Table 1).

**Table 1:** Validation methods applied to Maxent Species Distribution Models for *Rhinolophus euryale* and *R. mehelyi.*

Model	AUC Training	SD	AUC Test	SD	AUC_diff_	SD	TSS	SD
Current								
SAR	0.965	0.005	0.932	0.021	0.033	0.024	0.680	0.165
PES projected to Sardinia	0.997	0.000	0.993	0.003	0.004	0.003	0.804	0.170
*R. mehelyi* Sardinia	0.863	0.018	0.782	0.040	0.082	0.057	0.523	0.109
LGM								
SAR CCSM	0.944	0.004	0.926	0.014	0.018	0.022	0.768	0.065
SAR MIROC	0.987	0.006	0.917	0.016	0.070	0.005	0.754	0.045
PES CCSM	0.884	0.002	0.854	0.005	0.030	0.015	0.844	0.022
PES MIROC	0.885	0.003	0.877	0.002	0.008	0.022	0.799	0.056

SAR = Sardinian population of *R. euryale*; PES = Populations of *R. euryale* of Peninsular Italy and Sicily.

**Figure 2:**
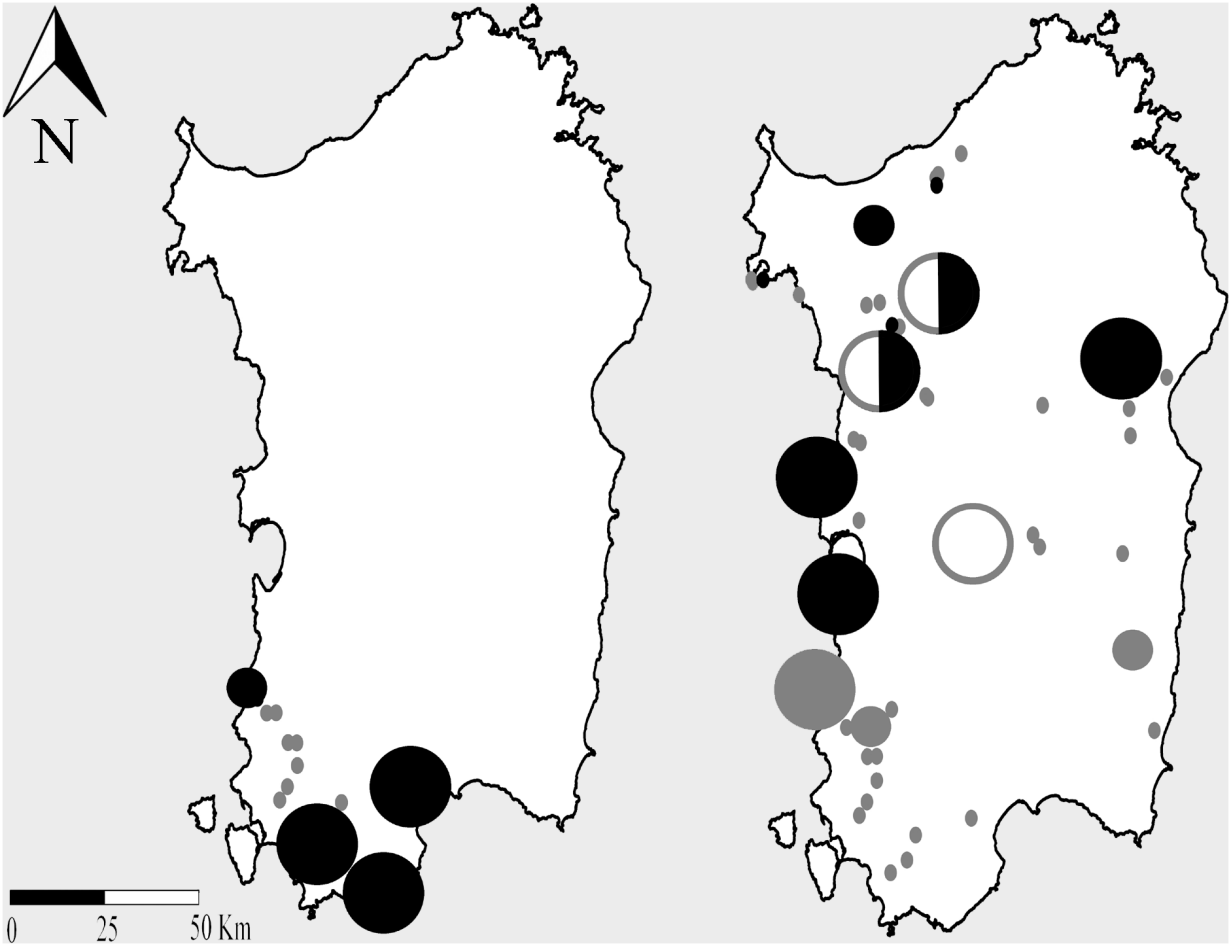
Distribution and colony size on Sardinia for *Rhinolophus euryale* (left) and *R. mehelyi* (right). Circle sizes are proportional to colony sizes. Black: nursery colonies; white: hibernacula; grey: other day-roost. Mixed-colour (white + black) symbols correspond to sites used by bats year round for both hibernation and reproduction. The publicly available map layer was obtained from www.fao.org/geonetwork/srv/en/main.home and the image prepared with the Quantum Gis 2.2.0 Valmiera open source software.

**Figure 3:**
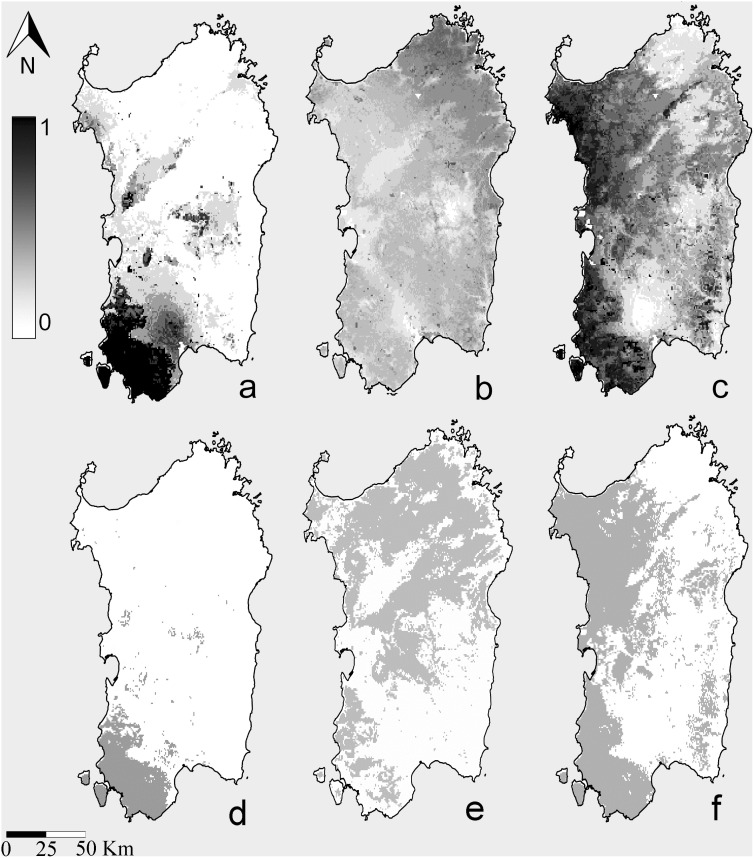
Maxent Species Distribution Models (SDM). a: SDM for *R. euryale* on Sardinia calibrated with Sardinian records only; b: SDM for *Rhinolophus euryale* on Sardinia calibrated with presence records from Italian populations except that of Sardinia and projected to the island; c: SDM for *R. mehelyi* on Sardinia calibrated with Sardinian records only; c: binary map for *R. euryale* on Sardinia calibrated with Sardinian records only; d: binary map for *Rhinolophus euryale* on Sardinia calibrated with presence records from Italian populations except that of Sardinia and projected to the island; e: binary map for *R. mehelyi* on Sardinia calibrated with Sardinian records only The publicly available map layer was obtained from www.fao.org/geonetwork/srv/en/main.home and the image prepared with the Quantum Gis 2.2.0 Valmiera and Maxent open source software packages.

Distributional data showed that the two species are sympatric only in the southern portion of the island, to which *R. euryale* is confined (Figure 2). This distribution matches the prediction made by Maxent model for SAR (Figure 3). Besides, large colonies of *R. mehelyi* are found in most of the island (where only this bat, but not *R. euryale* occurs) except in the restricted area where *R. euryale* is present: there *R. mehelyi* only occurs with small numbers (Figure 2).

Maximum entropy models trained with PES presence data and projected to Sardinia show that according to PES ecological requirements, *R. euryale* would occupy a larger area and, compared to SAR, have a reduced probability of presence in the southern portion of the island (Figure 3). This distributional pattern would determine larger areas of sympatry with *R. mehelyi*, as also shown by Maxent’s prediction for this species (Figure 3) hence increasing the likelihood of competition. From binary maps it can be derived that the predicted range overlap between *R. mehelyi* and PES is ca. 60%, while the former overlaps with SAR only by ca. 20% (Figure 3). The competition scenario is also supported by the fact that six out of nine niche analysis methods showed a significant similarity of *R. mehelyi* with PES (Table 2). Only two methods supported the similarity of PES with *R. mehelyi* (Table 2).

**Table 2:** Schoener’s D and Niche similarity significance levels relative to ordination methods and Species Distribution Models used to carry out niche comparison.

Comparison	Method	Schoener’s D	Niche Similarity	
*R. mehelyi* vs. SAR			SAR → *R. mehelyi*	*R. mehelyi* → SAR
	Between group	0.473	0.039+	0.019+
	LDA	0.561	0.019+	0.019+
	Maxent1	0.594	0.019+	0.019+
	Maxent2	0.628	0.019+	0.019+
	MDS	0.205	0.019+	0.950
	PCA environmental	0.215	0.059	0.495
	PCA Occurrence	0.229	0.019+	0.099
	Within environmental	0.215	0.039+	0.455
	Within group	0.127	0.079	0.871
SAR vs. PES			PES → SAR	SAR → PES
	Between group	0.000	2.000	2.000
	LDA	0.000	2.000	2.000
	Maxent1	0.014	2.000	2.000
	Maxent2	0.014	0.792	0.019−
	MDS	0.000	2.000	1.584
	PCA environmental	0.000	2.000	2.000
	PCA Occurrence	0.000	0.673	0.495
	Within environmental	0.124	0.970	0.119
	Within group	0.124	0.891	0.733
*R. mehelyi* vs. PES			PES → *R. mehelyi*	*R. mehelyi* → PES
	Between group	0.409	0.554	0.019+
	LDA	0.060	0.831	0.198
	Maxent1	0.190	0.039+	0.415
	Maxent2	0.215	0.376	0.534
	MDS	0.103	0.594	0.019+
	PCA environmental	0.176	0.099	0.019+
	PCA Occurrence	0.170	0.178	0.019+
	Within environmental	0.216	0.396	0.019+
	Within group	0.243	0.039+	0.019+

LDA = Linear Discriminant Analysis; Maxent 1 and 2 = Maximum Entropy Algorithm analysis made using in turn one of the two Maxent outputs generated by either population (or species) as the comparison background against which the output of the remainder was contrasted; MDS = Multidimensional scaling; PCA environmental = Principal Component Analysis calibrated on the whole environmental space including the presence records where the species occur; PCA occurrence = Principal Component Analysis calibrated with EGV values associated with the occurrences of the species; Within environmental = Within-group calibrated on the whole environmental space. + = similarity; − = dissimilarity.

**Figure 4:**
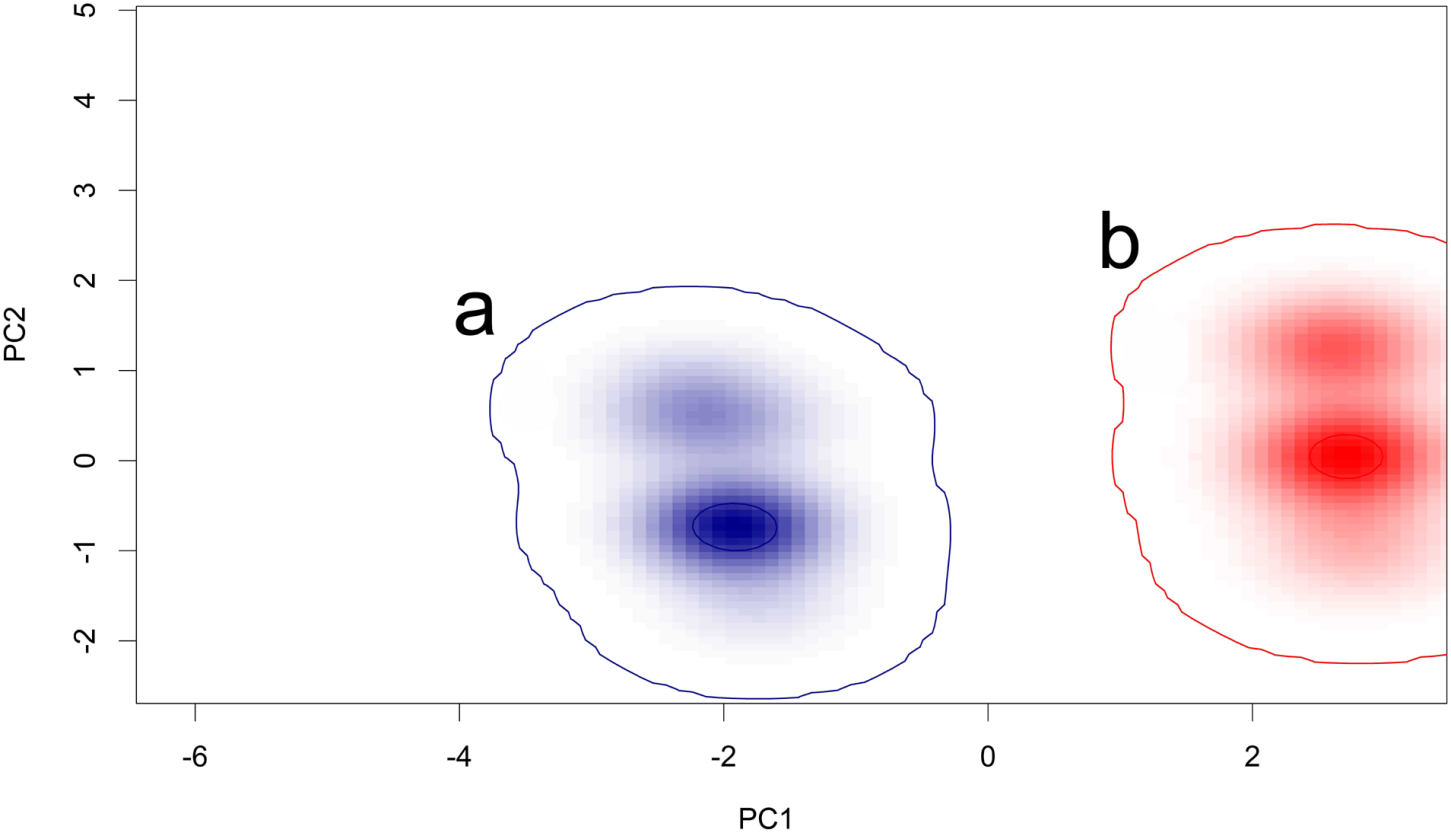
Graphical representation of the environmental niches for *Rhinolophus euryale*. a: Sardinian population; b: other Italian populations. In the example, niche were generated with Principal Component Analysis calibrated on the whole environmental space including the presence records where the species occur.

Niche comparison between SAR and PES showed no significant similarity (Table 2; Figure 4). The climatic variables that were most important to explain the potential distribution of SAR and PES were different. SAR is mainly localized in areas characterized by high isothermality values, mean temperature of wettest quarter of ca. 10–11°C, low standard deviation values of temperature seasonality and mean diurnal range between 8.5°C–10.5°C. SAR is also more likely to occur in areas of bare ground and mixed-leaved woodland at lower altitude. Such characteristics are found in SW Sardinia where SAR occurs. Suitability for PES decreases with increasing temperature seasonality. In the areas where the species’ likelihood of occurrence is high (central and northern Sardinia), the mean diurnal temperature range is ca. 8°C and % precipitation seasonality is low.

Niche comparison carried out for *R. mehelyi* vs. SAR showed a substantial similarity, supported respectively by 7 (*R. mehelyi* vs. SAR) and 4 (SAR vs. *R. mehelyi*) methods in either direction (Table 2). As found for PES, *R. mehelyi* probability of occurrence on Sardinia decreased for increasing values of temperature seasonality (Figure S1) and was also associated to wooded habitats.

Overall, the analysis supports the existence of niche divergence between SAR and PES and shows that this results in a smaller overlap between the ranges of *R. mehelyi* and *R. euryale* on the island.

### Niche difference as a legacy of biogeographic origin?

**Figure 5:**
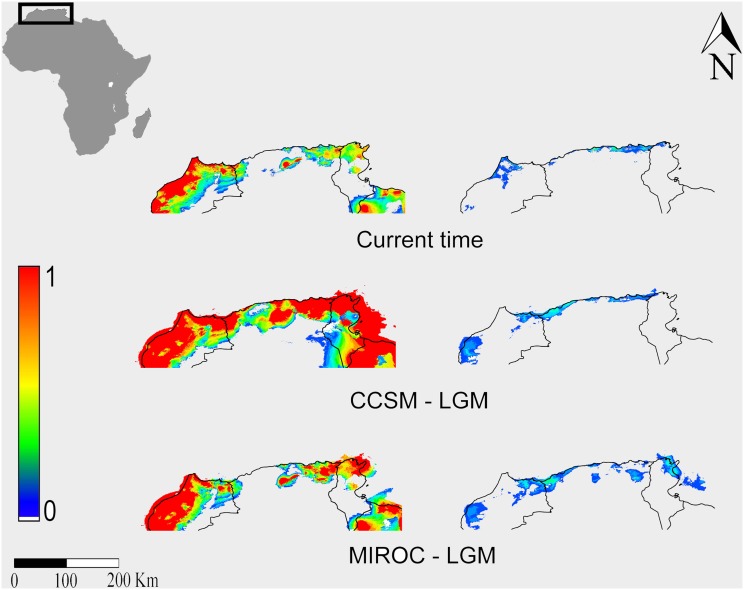
Maxent SDMs for *Rhinolophus euryale* calibrated respectively on presence records of bats from Sardinia (left) and from the remaining Italian areas (right) and projected to northern Africa. LGM = Last Glacial Maximum. CCSM = Community Climate System Model; MIROC = Model for Interdisciplinary Research on Climate. The publicly available map layer was obtained from www.fao.org/geonetwork/srv/en/main.home and the image prepared with the Quantum Gis 2.2.0 Valmiera and Maxent open source software packages.

Palaeo-distribution models too showed excellent levels of predictive performance as can be seen from AUC, TSS and AUC_diff_ values (Table 1). Our reconstruction showed that both in the present time and under the LGM scenario SAR would largely occur in northern Africa whereas PES would be practically absent, in agreement with a Maghrebian origin of SAR (Figure 5).

## Discussion

### Niche differences between R. mehelyi, SAR and PES

We showed that *R. euryale* on Sardinia is confined to a small southern portion of the island where it occurs in sympatry with *R. mehelyi* although in that area the latter is far less numerous than in the north, where *R. euryale* is absent. The niches of PES and *R. mehelyi* are similar but PES and SAR niches are not. If SAR had shown a niche identical to that of PES, the geographical distribution of *R. euryale* and *R. mehelyi* on the island would be largely sympatric, potentially leading to stronger interspecific competition. We conclude that niche differences between *R. mehelyi* and SAR minimize sympatry and thus potential competition. This result is consistent with the hypothesis that Sardinian *R. euryale* experienced a niche displacement process to mitigate competition with the numerically dominant *R. mehelyi*. Based on our models, the probabilities of occurrence of both *R. mehelyi* and SAR in the north-east sector of the island are small, whereas PES shows higher values. Why *R. euryale* has not occupied that region where competition would be low (at least based on PES ecological characteristics) appears less clear and perhaps explained by the biogeographic origin of Sardinian *R. euryale* (discussed below). Besides, based on the niche analysis results, the competition hypothesis would not be fully supported since despite the separation of SAR and PES niches, the former still partly overlaps with that of *R. mehelyi*. This may be explained by the fact that species distribution models were built from occurrence records but did not take colony size into account. Survey data instead showed that *R. mehelyi* occurs with large colonies in the areas of allopatry with SAR, but where the two species are sympatric, it is only present with smaller numbers. In other words, modelling based on presence records probably overestimated niche similarity by disregarding local population size: the difference between the two niches of Sardinian *R. mehelyi* and *R. euryale* may thus be even larger than that estimated here.

Whatever the reason for its peculiarity, SAR niche must have allowed *R. euryale* to establish a viable population in an area where *R. mehelyi* appears to perform less well and thus be less competitive, as can be inferred from the smaller colony sizes of the latter in the southern area of sympatry. Noticeably, the two species are known to share roosting sites in their Mediterranean regions of sympatry [38], including Sardinia (this study). Bats often form mixed-species groups when they have common thermal preferences, and interspecific associations may result in mutual thermoregulatory ( = energetic) benefits [62]. Based on such considerations, we rule out that the species we considered compete for roosting sites.

Food is much more likely to trigger competition. The diet of both species is well known, and is mostly made of moths both in sympatry and allopatry; the amounts of other prey only show small interspecific differences [35], [63], [64] In most areas of sympatry but not on Sardinia, echolocation call frequencies of *R. mehelyi* and *R. euryale* largely overlap each other [35], [38], [65] leading to the detection of similar prey [35], [38]. Although Sardinian *R. euryale* have lower frequencies than local *R. mehelyi* [38], the difference is too small to account for niche partitioning. Since echolocation calls also convey individual information among conspecifics [66], [67] this difference is best explained as a way to maintain separate communication bandwidth in the area of sympatry [38].

Foraging habitat use in these rhinolophids shows no interspecific differences in allopatric populations [35] but does differ in sympatry, where *R. mehelyi* performs better in less structurally complex habitats than in more closed vegetation [35], [36] due to its lower flight manoeuvrability and agility [35], [36], [68]. Over the millennia, hundreds of human generations have shaped Sardinian landscapes and microclimates through deforestation, stock breeding and fires [69] so that much of the land is covered with Mediterranean scrubland and open forest, where *R. mehelyi* is probably more competitive than *R. euryale*. Reduced habitat heterogeneity such as that found on Sardinia as well as the limitedness of food, typical of insular systems [70] can be important factors increasing competition between the two rhinolophids.

### Niche difference as a legacy of biogeographic origin?

In principle, our findings are in agreement with a niche displacement process: in fact, one possible scenario is that Sardinia was colonized by *R. euryale* from mainland Europe and that the newly established population shifted its ecological niche to counter competition pressures from heterospecific bats (*R. mehelyi*). However, questions arise on the geographical source of Sardinian *R. euryale* and its ecological consequences as an alternative explanation for SAR’s niche distinctness. *R. euryale* might have colonized Sardinia from the Maghreb and the peculiar ecological niche of Sardinian bats could thus be a legacy of the African source population rather than the outcome of a niche displacement process. This niche may have been subjected to stabilizing selection [44] and conserved as it must have performed well to allow co-existence with insular *R. mehelyi*. By projecting respectively SAR and PES niches to the Maghreb both in the current time and in the LGM we found a striking difference in the probability of occurrence, much higher for SAR’s projection. This result is in agreement with a possible SAR’s Maghrebian origin.

Stretches of sea have been found to represent barriers to the movement of bats [71], [72] although their permeability differs across species. The capacity of different species to overcome such barriers is not related to wing morphology and flight performances [73]. Colonization events would seem as difficult from mainland Italy as they would be from northern Africa. Sardinia lies ca. 200 km off the coasts of both regions, so both routes appear equally likely to explain the origin of Sardinian *R. euryale*. Colonization of Sardinia by bats was only possible across the sea since the end of the Messinian Event, ending 5.33 million years ago [43], [74]. Thus, if SAR had an African origin, its establishment would either date back to the Messinian Event or, if more recent, must have implied crossing the sea. The latter option is possible: there is evidence that after the Messinian Event Sardinia was subject to repeated bat colonization waves at different times (including recent ones) from Europe and northern Africa, as for the Maghrebian bat *Myotis punicus* [42], long-eared bats [41] and pipistrelles [43]. Glacial episodes that repeatedly occurred in the Pleistocene lowered sea levels and led to the emersion of land bridges [45], interrupting the isolation of Sardinia from the mainland during early and mid-Pleistocene, and favoured island colonization most probably via a stepping stone geographic system. This would be in agreement with the wide northern African distribution we obtained for *R. euryale* during the LGM by projecting SAR’s niche to that region. Caution is needed when considering our LGM models for the Sahara area, where some overpredictions occurred. These were most likely due to the lack of solid information on the region’s climate at that age [75] inevitably affecting the reliability of climatic variables.

Although these findings do not prove the biogeographical origin of SAR, we hope they will stimulate molecular studies investigating the phylogeography of *R. euryale* in the Mediterranean Basin for a final answer on the identity of SAR’s geological source and a full reconstruction of this population’s history.

## Supporting Information

Figure S1 **EGV response curves of Maxent SDMs for selected ecogeographic variables.** a: Temperature seasonality for *R. mehelyi*; b: Temperature seasonality for PES; c: Isothermality for SAR.
(TIF)

Table S1 **List of ecogeographical variables used for this study, their type and measurement unit.**
(DOC)

File S1 **Description of Niche Analysis.**
(DOC)
