# Correction: What Story Does Geographic Separation of Insular Bats Tell? A Case Study on Sardinian Rhinolophids

**DOI:** 10.1371/journal.pone.0115718

**Published:** 2014-12-16

**Authors:** The PLOS ONE Staff

There is an error in the legend of Figure 3. Please see the correct Figure 3 legend here.

**Figure 3:**
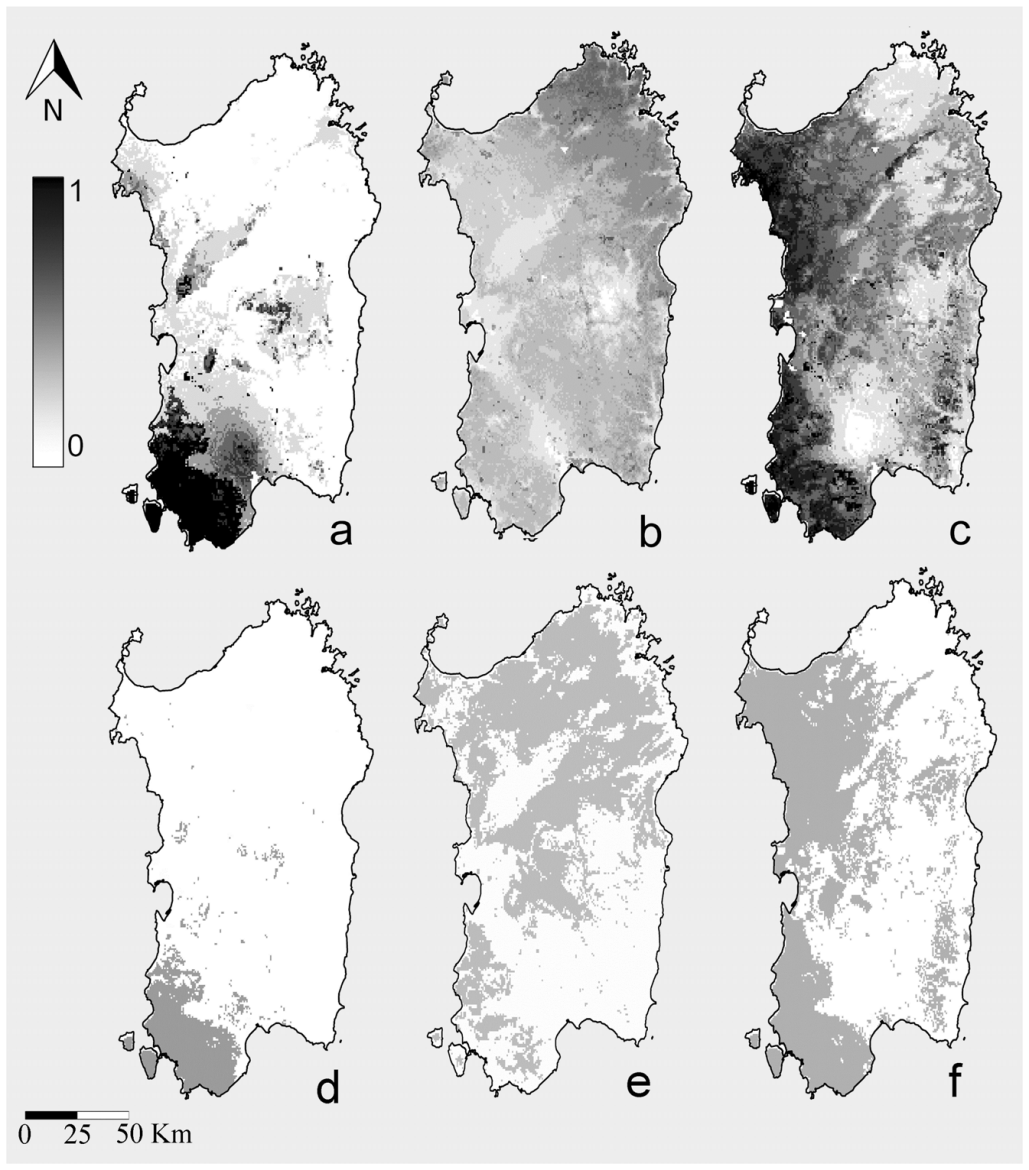
Maxent Species Distribution Models (SDM). a: SDM for *R. euryale* on Sardinia calibrated with Sardinian records only; b: SDM for *Rhinolophus euryale* on Sardinia calibrated with presence records from Italian populations except that of Sardinia and projected to the island; c: SDM for *R. mehelyi* on Sardinia calibrated with Sardinian records only; d: binary map for *R. euryale* on Sardinia calibrated with Sardinian records only; e: binary map for *Rhinolophus euryale* on Sardinia calibrated with presence records from Italian populations except that of Sardinia and projected to the island; f: binary map for *R. mehelyi* on Sardinia calibrated with Sardinian records only The publicly available map layer was obtained from www.fao.org/geonetwork/srv/en/main.home and the image prepared with the Quantum Gis 2.2.0 Valmiera and Maxent open source software packages.
