# Supplementary figures and images for: What Story Does Geographic Separation of Insular Bats Tell? A Case Study on Sardinian Rhinolophids
Source: PLoS One. 2014 Oct 23;9(10):e110894. doi: 10.1371/journal.pone.0110894 (PMC4207767; doi:10.1371/journal.pone.0110894)

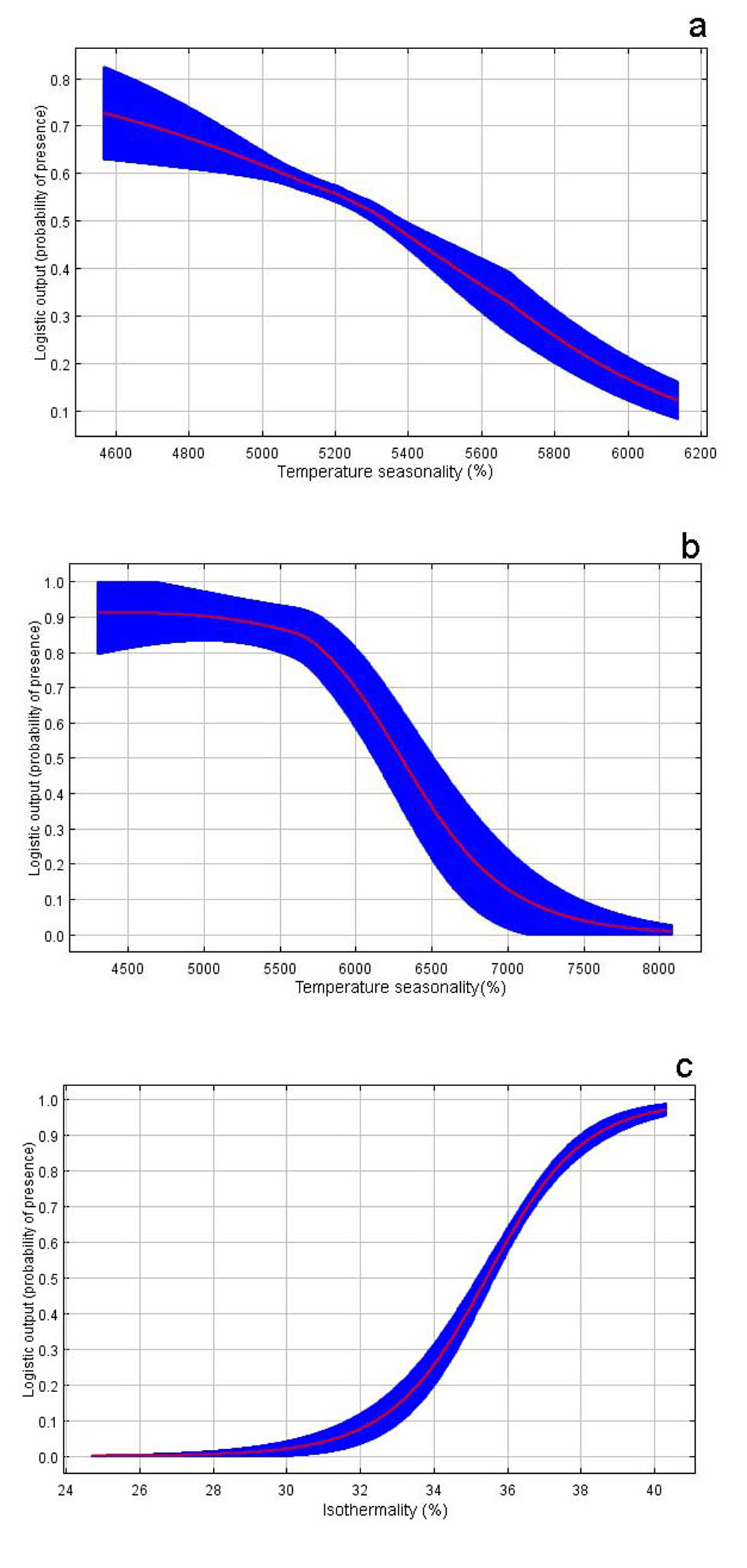

Supplement: Figure S1 — EGV response curves of Maxent SDMs for selected ecogeographic variables. a: Temperature seasonality for R. mehelyi; b: Temperature seasonality for PES; c: Isothermality for SAR. (TIF) [file pone.0110894.s001.tif]
