# Supplementary material for: What Story Does Geographic Separation of Insular Bats Tell? A Case Study on Sardinian Rhinolophids
Source: PLoS One. 2014 Oct 23;9(10):e110894. doi: 10.1371/journal.pone.0110894 (PMC4207767; doi:10.1371/journal.pone.0110894)
Supplement: Table S1 — List of ecogeographical variables used for this study, their type and measurement unit. (DOC) [file pone.0110894.s002.doc]

| Type | Ecogeographical Variable | Unit |
| --- | --- | --- |
| Topographical | Altitude | m |
| Habitat | Land cover | - |
| Climatic | Mean Diurnal Range | °C |
|  | Isothermality | % |
|  | Temperature Seasonality | °C |
|  | Temperature Annual Range | °C |
|  | Mean Temperature of Wettest Quarter | °C |
|  | Mean Temperature of Driest Quarter | °C |
|  | Precipitation Seasonality | % |
|  | Precipitation of Wettest Quarter | mm |
|  | Precipitation of Coldest Quarter | mm |

Table S1. List of ecogeographical variables used for this study, type and measurement unit. For further details please refer to:

http://pubs.usgs.gov/ds/691/ds691.pdf
