# Supplementary material for: What Story Does Geographic Separation of Insular Bats Tell? A Case Study on Sardinian Rhinolophids
Source: PLoS One. 2014 Oct 23;9(10):e110894. doi: 10.1371/journal.pone.0110894 (PMC4207767; doi:10.1371/journal.pone.0110894)
Supplement: File S1 — Description of Niche Analysis. (DOC) [file pone.0110894.s003.doc]

Supporting information S2

*Niche analysis*

i) Data pre-processing. The environmental space, as obtained from a multivariate ordination or from the prediction of a species distribution model is divided in cells, each corresponding to a unique vector of environmental conditions ν_ij_, observed at one or more sites in the geographical space. A Kernel density function [1] is then applied to calculate the density of number of occurrences and number of sites with particular environmental conditions ν_ij_, for each cell of the environmental space. Such densities are divided respectively by the maximum number of occurrences in all cells of the environmental space, max(n_ij_), and by the number of sites with the commonest environment, max(N_ij_), as follows:

$o_{ij}=\frac{\delta\left(n_{ij}\right)}{\max\left(n_{ij}\right)}$, $e_{ij}=\frac{\delta\left(N_{ij}\right)}{\max\left(N_{ij}\right)}$ (1)

*o*_ij_ and *e*_ij_ are two indices ranging from 0 – for environments with neither occurrences nor sites – to 1 – for environments with maximum numbers of occurrences and sites. Then *z*_ij_, the occupancy of each environment ν_ij_ by the species, can be calculated as follows:

$z_{ij}=\frac{o_{ij}/e_{ij}}{\max\left(o/e\right)}$, if $e_{ij}\neq0$ (2)

$z_{ij}=0$, if $e_{ij}=0$

This index also ranges from 0 to 1 and makes it possible to catty out unbiased comparisons of occurrence densities between different entities found in ranges with environments not equally available [2].

This data pre-processing step avoids problems due to the spatial resolution of the data, corrects densities of observed occurrence for each region in light of the availability of environmental space and most importantly, optimizes the use of both geographical and environmental spaces.

ii) Calculation of the niche overlap measure. In this step the niche overlap in the environmental space is measured, using a revised version of the *D* metric [3], as follows:

(3)

*D* metric offers a calculation of niche overlap between two species by comparing their occupancy values, *z*_ij_, calculated in equation 2, over the environmental space.

iii) Testing similarity [3]. The similarity test relies on a randomization process evaluating how many similarities or dissimilarities between the niches of two species may be attributed to ecological issues (habitat selection and/or suitability) or is rather due to chance. The test involves generating a null distribution of 100 simulated niche overlap values by shifting randomly the centre of the observed distribution of occurrences in one species’ range and calculating niche overlap values between the simulated niches and the observed niche calculated in the other species’ range (range 1 🡪 range 2). The procedure is also done out in the opposite direction, simulating niches in range 2 and calculating niche overlap with observed niche in range 1 (range 1 🡨 range 2). If the observed niche overlap value is larger or smaller than the null distribution of the simulated values, the two niches are more similar/different than expected by chance [2,3].

*References*

1. Silverman BW (1986) Density estimation for statistics and data analysis. In: Chapman and Hall, editors. Monographs on Statistics and Applied Probability. London: United Kingdom. pp. 1 – 22.

2. Broennimann O, Fitzpatrick MC, Pearman PB, Petitpierre B, Pellissier L, Yoccoz NG, Yoccoz NG, Thuiller W, Fortin MJ, Randin C, Zimmermann NE, Graham CH, Guisan A (2012) Measuring ecological niche overlap from occurrence and spatial environmental data. Global Ecology and Biogeography 21**:** 481–497.

3. Warren DL, Glor RE, Turelli M (2008) Environmental niche equivalency versus conservatism: quantitative approaches to niche evolution. Evolution 62**:** 2868–2883.
